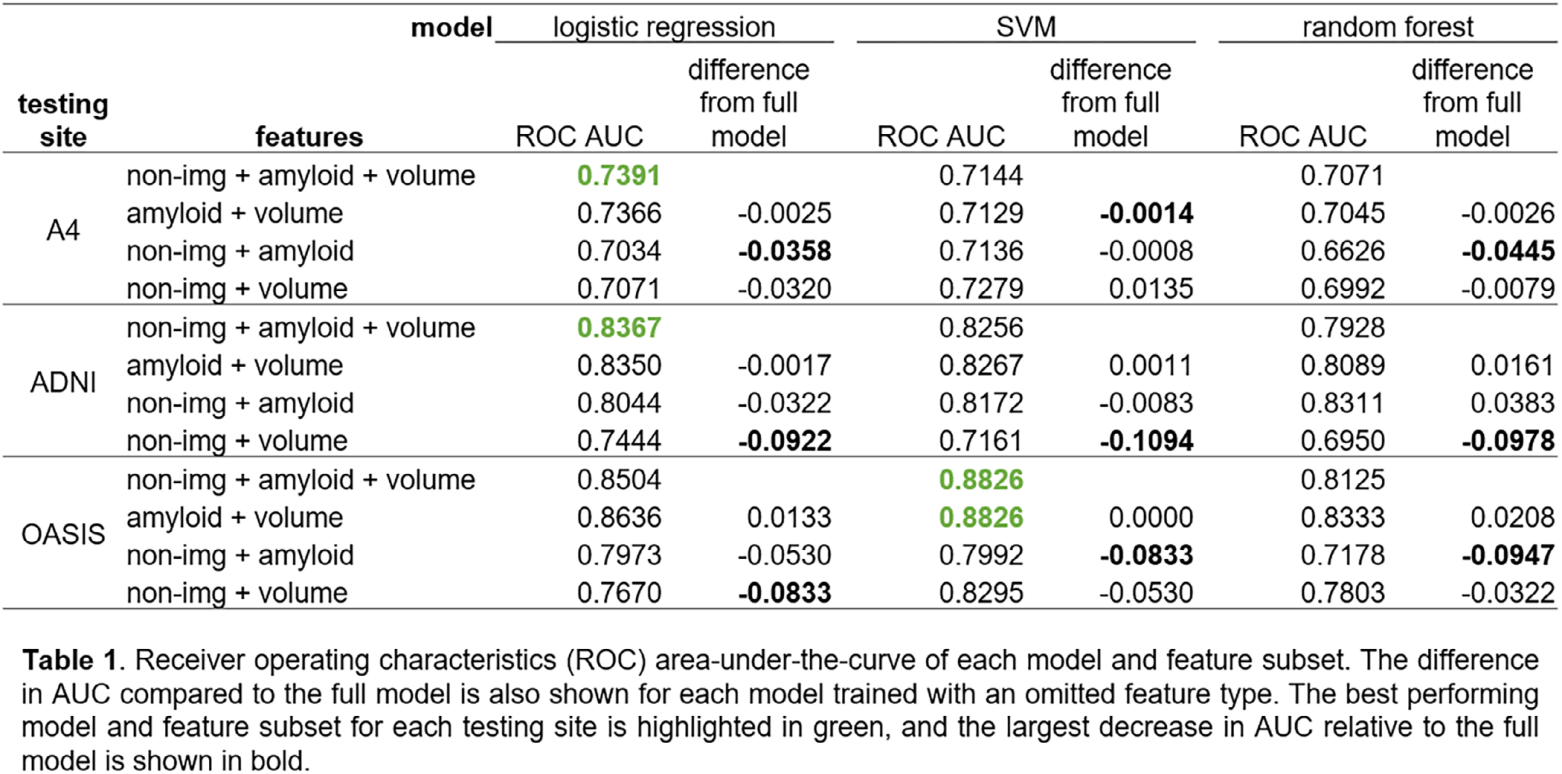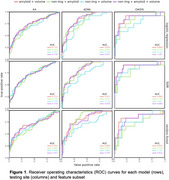# A multisite evaluation of machine learning classifiers to predict progression to mild cognitive impairment using multimodal imaging

**DOI:** 10.1002/alz70856_107362

**Published:** 2026-01-09

**Authors:** Braden Yang, Tom Earnest, Murat Bilgel, Tammie L.S. Benzinger, Brian A. Gordon, Aristeidis Sotiras

**Affiliations:** ^1^ Mallinckrodt Institute of Radiology, Washington University School of Medicine in St Louis, St Louis, MO, USA; ^2^ Laboratory of Behavioral Neurosciences, National Institute on Aging Intramural Research Program, National Institutes of Health, Baltimore, MD, USA; ^3^ Mallinckrodt Institute of Radiology, Washington University School of Medicine, St. Louis, MO, USA; ^4^ Institute for Informatics, Washington University, St. Louis, MO, USA

## Abstract

**Background:**

Predicting progression to mild cognitive impairment (MCI) and dementia in preclinical AD patients is crucial for proper recruitment into anti‐amyloid clinical trials. We evaluated the predictive ability of machine learning (ML) classifiers for distinguishing MCI‐progressors from non‐progressors using baseline amyloid positron emission tomography (PET) and magnetic resonance imaging (MRI) features as predictors.

**Method:**

We selected cognitively‐normal, amyloid‐positive participants from ADNI (*N* = 86, 36 progressors), OASIS (*N* = 56, 12 progressors), and A4 (*N* = 210, 79 progressors). Subjects were classified as stable (remain CDR=0 at least 3 years from baseline) or progressor (convert to CDR>0 within 3 years of baseline). Each subject's first amyloid‐positive [^18^F]‐florbetapir PET scan and matching T1‐weighted MRI underwent standard PET‐MRI processing to obtain 86 regions‐of‐interest, from which regional standardized uptake value ratios and volume were computed. Principal component analysis was applied to reduce the number of amyloid and volume features. Age, sex, and APOE‐e4 carriership were also included as predictors. Three ML classifiers – logistic regression, support vector machine (SVM), and random forest – were trained to predict binary stable/progressor class. Data from two sites were used to train models and optimize hyperparameters using 5‐fold cross‐validation, while the third site was held out for testing. Performance was quantified using receiver operating characteristics area‐under‐the‐curve (AUC). To assess the importance of each predictor type (non‐imaging, amyloid, volume), nested models were trained by omitting one predictor type, and its AUC was compared to the model trained on all predictor types.

**Result:**

Logistic regression with the full set of features performed the best with A4 (AUC=0.7391) and ADNI (AUC=0.8367) as the testing set, while SVM with either full features or with non‐imaging features omitted performed the best with OASIS as the testing set (AUC=0.8826) (Figure 1, Table 1). Omission of volumetric features generally resulted in the largest dip in AUC for A4 and OASIS testing sets, whereas omission of amyloid features resulted in the largest dip in AUC for ADNI (Table 1).

**Conclusion:**

ML classifiers utilizing multimodal imaging are predictive of progression to MCI in preclinical AD individuals and are robust to external testing sites.